# Microfluidic-Enabled Liposomes Elucidate Size-Dependent Transdermal Transport

**DOI:** 10.1371/journal.pone.0092978

**Published:** 2014-03-21

**Authors:** Renee R. Hood, Eric L. Kendall, Mariana Junqueira, Wyatt N. Vreeland, Zenaide Quezado, Julia C. Finkel, Don L. DeVoe

**Affiliations:** 1 Fischell Department of Bioengineering, University of Maryland, College Park, Maryland, United States of America; 2 Department of Mechanical Engineering, University of Maryland, College Park, Maryland, United States of America; 3 Department of Anesthesiology, Sedation, and Perioperative Medicine, Children's National Medical Center, Washington, DC, United States of America; 4 Biomolecular Measurement Division, National Institute of Standards and Technology, Gaithersburg, Maryland, United States of America; University of Illinois at Chicago, United States of America

## Abstract

Microfluidic synthesis of small and nearly-monodisperse liposomes is used to investigate the size-dependent passive transdermal transport of nanoscale lipid vesicles. While large liposomes with diameters above 105 nm are found to be excluded from deeper skin layers past the stratum corneum, the primary barrier to nanoparticle transport, liposomes with mean diameters between 31–41 nm exhibit significantly enhanced penetration. Furthermore, multicolor fluorescence imaging reveals that the smaller liposomes pass rapidly through the stratum corneum without vesicle rupture. These findings reveal that nanoscale liposomes with well-controlled size and minimal size variance are excellent vehicles for transdermal delivery of functional nanoparticle drugs.

## Introduction

Transdermal drug delivery offers significant potential as an alternative to oral delivery and hypodermic injection due to the promise of pain-free local or systemic introduction of drugs with controllable delivery rates over extended time periods [1]. Effective delivery of drug through the skin is hampered by poor diffusive transport across the stratum corneum (SC) layer, a 10 μm to 20 μm thick tissue region comprised of a structured lipid/protein matrix [2], [3]. Even when employing chemical penetration enhancers, a broad class of skin disrupting molecules including a variety of surfactants, [1], [4], transdermal drug delivery has met only limited success. Techniques such as dermabrasion and thermal ablation can temporarily render the SC porous to enhance drug transport, but these methods require active disruption of the skin and do not allow controlled doses to be delivered over long time periods. Similarly, non-invasive active methods such as ionophoresis and ultrasound require specialized equipment and only enhance drug transport for short periods. Nanoparticles offer an alternative strategy for passive transdermal delivery, offering increased drug loading, sustained release, and the potential for tissue-specific targeting. The structure of the SC includes lamellar lipid regions that present sub-nanometer intercellular spaces which can be widened in the presence of nanoparticle colloids to pores with dimensions on the order of several tens of nanometers [3], [5]. Inorganic quantum dots ranging from approximately 4 nm to 12 nm in diameter exhibit efficient passive transport across the SC [6], [7]. However, the utility of these nanoparticles for drug delivery is limited by high toxicity and low drug loading capacity [8], In contrast, lipid nanoparticles present a highly attractive route for drug delivery due to their excellent biocompatibility [9]. In particular, nanoscale liposomes with lipid bilayers encapsulating aqueous internal volumes offer high loading of both hydrophilic and amphipathic drugs, low toxicity, and tunable stability. However, there is little evidence that lipid vesicles ranging from 60 nm to several micrometers in diameter can traverse the SC in significant numbers [10]–[13], nor is there clear evidence of intact liposome passage through the SC. As a result, the application of lipid nanoparticles for transdermal drug delivery has largely focused on flexible liposomes such as transfersomes [14] and ethosomes [15], which incorporate surfactants or alcohols to impart a high degree of flexibility to the vesicle membranes, putatively allowing relatively large vesicles to traverse the small intercellular pores within the SC. However, for systemic delivery through the bloodstream, these nanoparticles are not ideal since large and flexible liposomes are subject to rapid opsonization and phagocytotic clearance. Furthermore, whereas both pharmacokinetics and biodistribution of traditional liposomes have been extensively studied and optimized, the behaviors of flexible liposomes remain largely unknown. More fundamentally, recent evidence indicates that ultraflexible transfersomes are highly compromised by passage through the skin, and may be no better than traditional liposomes for transdermal delivery of intact vesicles [16].

**Figure 1:**
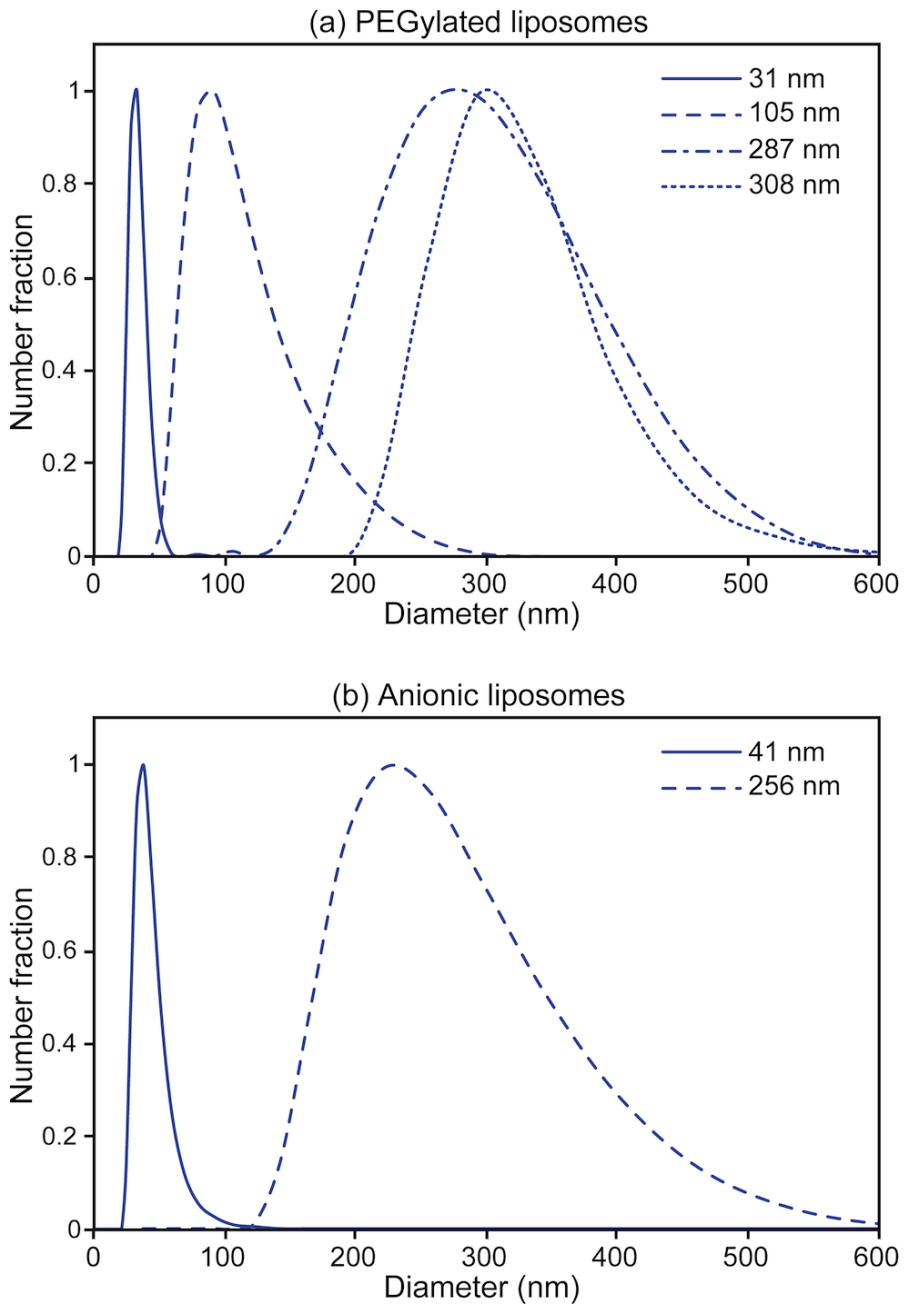
Volume-weighted size distributions of microfluidic-enabled (a) PEGylated and (b) anionic liposomes, revealing narrow size distributions over the full size range from 31 nm to 308 nm.

In this work we leverage a microfluidic technique that employs hydrodynamic focusing of a stream of solvated lipid sheathed by a sheath flow of aqueous buffer within a continuous-flow process [17]–[20]. This approach provides the ability to generate well-defined populations of small liposomes with narrow size distributions, enabling the effective study of the size-dependent transport of lipid vesicles across the SC. Conventional bulk methods of liposome production, including membrane extrusion [21] and sonication [22], are limited in their ability to generate well defined populations of liposomes with diameters in the size range expected to support effective transport of nanoparticles through the SC. As a result, prior studies have not shown extensive penetration of traditional liposomes past the SC [10]–[13]. In the present study, the capacity of the microfluidic technique to produce small liposomes with low polydispersity was exploited to generate populations of dye-laden vesicles that are almost entirely within the 25 nm to 40 nm diameter range previously reported to result in high transdermal flux of other monodisperse nanoparticles [23], [24]. Microfluidic-enabled liposome preparations with mean diameters ranging from 31 nm to 308 nm were prepared (Fig. 1). Within this size range, two classes of liposomes were formed that differed by the incorporation of small amounts of either anionic lipids or PEGylated lipids, enabling the influence of surface chemistry on trans-SC flux to be investigated. For all liposome preparations in this study, the polydispersity index of the microfluidic-synthesized liposomes ranged from 0.035 to 0.135; as a comparison, a previous study investigating vesicles as small as 120 nm reported the use of liposomes with polydispersity indices varying from 0.1 to 0.3 [25]. Overall, the microfluidic-enabled liposomes produced here are both smaller and more narrowly distributed in diameter than bulk-scale produced liposomes used in prior passive transdermal drug delivery studies. The use of smaller liposomes is significant due to the hypothesis that liposomes, like other nanoparticles [3], [5], [23], [24] will exhibit size-dependent dermal transport, with vesicles smaller than approximately 40 nm in diameter traversing the SC more effectively than larger nanoparticles. Similarly, the low polydispersity is significant since the total fluorescence signal from a vesicle population with a wide size distribution will be biased by the presence of significant number of liposomes above the mean diameter, prohibiting accurate evaluation of transport as a function of vesicle size. While a French press technique for liposome preparation has been reported to enable the formation of small unilamellar vesicles with diameters below approximately 30∼50 nm [26], [27], this method does not allow vesicle size to be readily tuned. The exceptionally low polydispersity of the microfluidic-enabled liposomes over a wide range of diameters allows a unique view into size-dependent dermal transport.

## Results and Discussion

**Figure 2:**
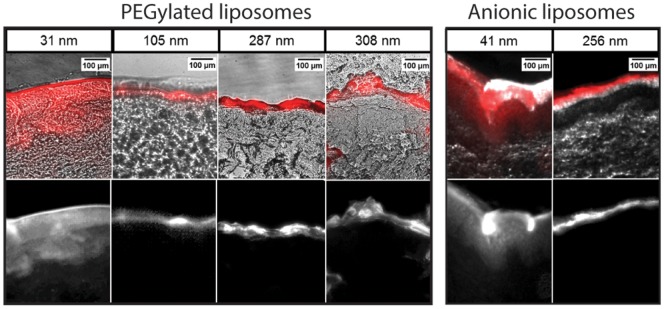
Brightfield/fluorescence image overlays (top) and single-channel fluorescence images (bottom) for microtomed tissue sections following 15 min application of PEGylated or anionic liposome samples of varying diameters containing DiI lipophilic dye. Significant dye penetration past the SC is observed with the smallest liposomes (31 nm diameter PEGylated and 41 nm anionic liposomes), while dye from the larger vesicles does not appear to cross the SC, indicating size-based passive transport independent of surface charge.

Fluorescence microscopy of microtomed porcine ear tissue after incubation with the various liposome preparations shows a marked difference in the dermal penetration of dyes between tissues exposed to either larger or smaller liposome preparations. Figure 2 shows representative fluorescent images revealing the distribution of DiI dye within the tissue sections. Skin samples exposed to the larger 105 nm to 308 nm liposomes (PEGylated and anionic) consistently exhibit bright bands of fluorescence associated with the SC, with very little fluorescence within deeper skin layers, revealing that these larger liposomes are either physically excluded by the narrow inter-corneocyte spaces, or are ruptured in the process of traversing the SC. In the latter case, the lipids and lipophilic dyes from the ruptured liposomes are likely to adhere to or associate with surrounding cells and extra-cellular material [28]. Conversely, the smaller 31 nm and 41 nm liposomes reveal a more evenly distributed dye profile throughout the skin, appearing to traverse the SC and enter the underlying layers of tissue in multiple instances with less significant accumulation in the SC (Fig. 2). We note that the several bright features that appear in deeper layers within some images are believed to result from imperfections caused by vessels or voids created during cryosectioning. Skin locations with capillaries were excluded due to the known autofluorescence of whole blood between wavelengths of 450–600 nm [29] and the relatively low concentration of fluorescent molecules in the liposome samples. Regions with significant voids created by tearing of the thin tissue sections during microtoming were excluded in order to maintain consistency throughout the samples. In control samples using free SF dye applied to the skin in liposome-free buffer, no penetration beyond the SC was observed. Dye penetration into deeper skin layers shows a strong dependence on liposome size, irrespective of charge state as determined by the presentation of PEG or anionic lipids on the vesicle surfaces. This is consistent with the hypothesis that dermal transport of lipid vesicles is a size-based phenomenon, and the ability of the smallest liposomes to traverse the SC and reach lower layers of skin is a direct result of the reduced liposome diameters. In some samples, bright and highly localized defects were visible in the dermis and epidermis. These features are routinely observed in dermal transport studies, and are the result of enhanced particle transport through hair-follicles, pores, and skin perforations [30], [31]. This uneven, defect-based liposome penetration pathway is, by nature, not highly correlated to liposome size [30], [31]. The more diffuse, evenly distributed fluorescence signal seen in the epidermis in the small (31 nm and 41 nm) liposome samples is evidence of liposome transport across the SC by a passive inter-corneocyte pathway, a similar phenomenon seen with other nanoparticles below 40 nm in diameter [3], [5], [23], [24]. For tissue samples where hair follicles were present, enhanced transport was observed for all liposomes populations. Results from these samples were omitted from analysis to prevent the confounding influence of follicular transport on analysis of SC penetration.

**Figure 3:**
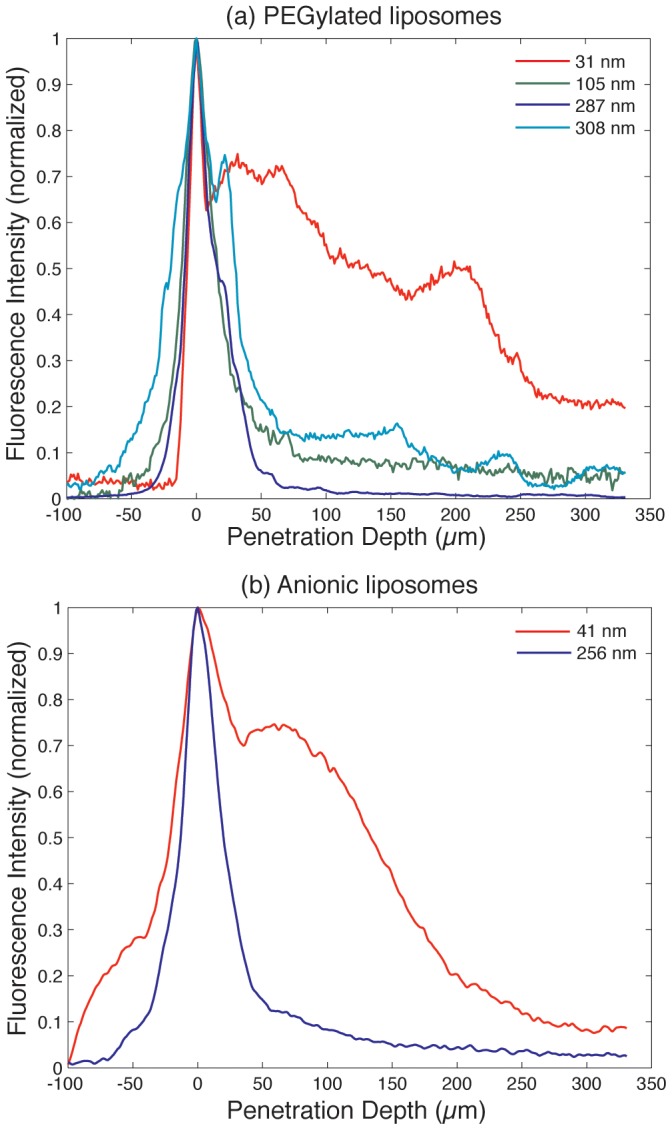
DiI fluorescence intensity plot profiles for (a) PEGylated liposomes and (b) anionic liposomes as a function of porcine skin tissue penetration depth. Measurements were performed 15 minute following liposome application. Each curve is representative of an average of 5 ROIs per image.

**Figure 4:**
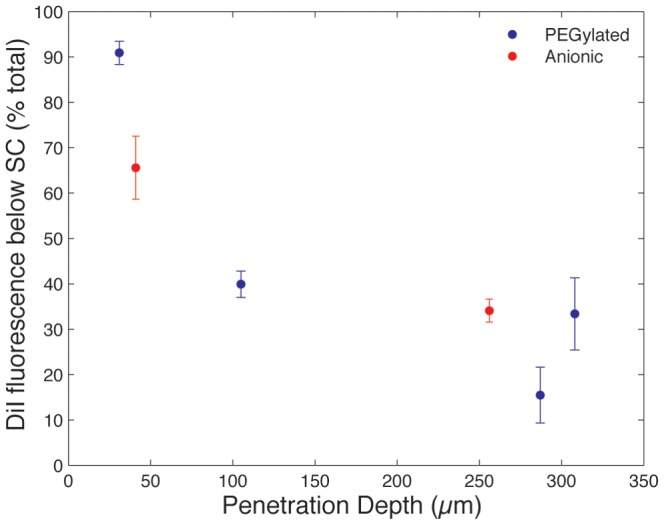
Percentage of total DiI fluorescence signal seen below the SC for the different sizes of PEGylated and anionic liposomes. Each plot reflects the average profile extracted from 5 ROIs per tissue section, with error bars reflecting standard deviation. SC thickness, estimated from averaged manual measurements using brightfield images of each tissue, ranged from 15 μm to 40 μm, in general agreement with previously reported values for porcine skin [32]. The small 31 nm PEGylated liposomes pass the SC in large numbers (91%), which is up to 590% greater than the larger 105 nm to 308 nm diameter liposomes. The small 41 nm anionic liposomes also reveal 65% of their total DiI signal under the SC, which is 200% greater than observed with 256 nm diameter liposomes of the same composition.

For quantitative comparison of liposome penetration, ImageJ software was used to obtain plot profiles of fluorescence intensity normal to the tissue surface. Profiles of each tissue section were averaged over 5 representative regions per sample (Fig. 3). These profiles were normalized for maximum fluorescence intensity per profile and aligned to the midpoint of the SC, across all samples. The SC thickness was determined from averaged manual measurements using brightfield images of each tissue, ranging from 15 μm to 40 μm, which is in agreement with previously reported values for porcine skin [32]. The percentage of DiI fluorescence intensity observed beneath the SC compared to the total observed fluorescence signal was calculated from the plot profiles for each sample and compared across different liposome sizes and surface chemistries (Fig. 4). We note that this technique assumes a linear relationship between fluorescence intensity and liposome concentration, an assumption that does not hold for samples where liposomes are highly concentrated in one area causing a local saturation of fluorescence intensity, as observed in some images from the larger (diameter greater than 105 nm) liposomes used in this study. This saturation effect leads to systematic underreporting of liposomes trapped in the SC, and thus a bias toward higher measured penetration efficiencies for these larger liposomes can occur. Detector saturation was avoided as much as possible while maintaining identical imaging conditions across all samples used in this study. We also note that while efforts were made to omit from analysis tissue sections with large voids, blood vessels, or hair follicles, some regions with anomalous fluorescent patches do appear in several images, particularly for the larger 308 nm PEGylated liposomes as seen in Fig. 2.

The small 31 nm PEGylated liposomes pass the SC in large numbers (91%), which is up to 590% greater than the larger 105 nm to 308 nm vesicles studied here. The small 41 nm diameter anionic liposomes show 65% of their total DiI signal under the SC, which is 200% greater than observed with 256 nm diameter liposomes. Both populations of smaller liposomes exhibit significantly enhanced penetration through dermal tissues compared to the larger vesicles, which is consistent with the behavior observed for other nanoparticles smaller than 40 nm in diameter [23], [24], and reveals that size-dependent transdermal transport of the microfluidic-enabled liposomes follows the same overall trend observed for other classes of nanoparticles.

**Figure 5:**
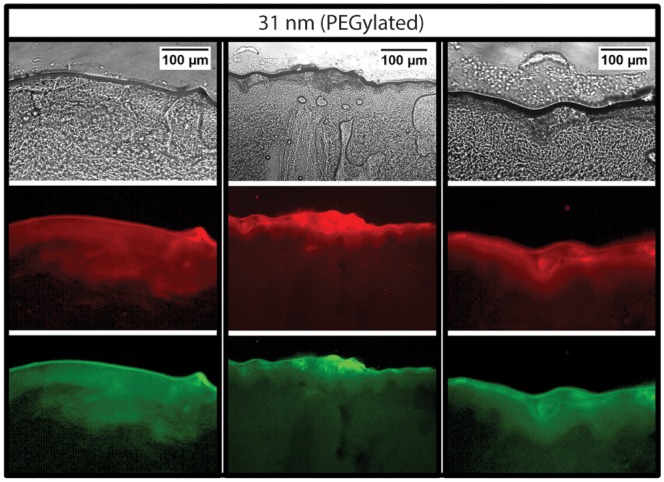
Brightfield images of 3 representative tissue regions following application of 31(top), together with matched single channel fluorescence images for lipophilic DiI (middle) and hydrophilic SF (bottom). Similar fluorescence distributions for both dyes are seen across multiple tissue sections, indicating successful penetration of intact liposomes through the epithelium.

**Figure 6:**
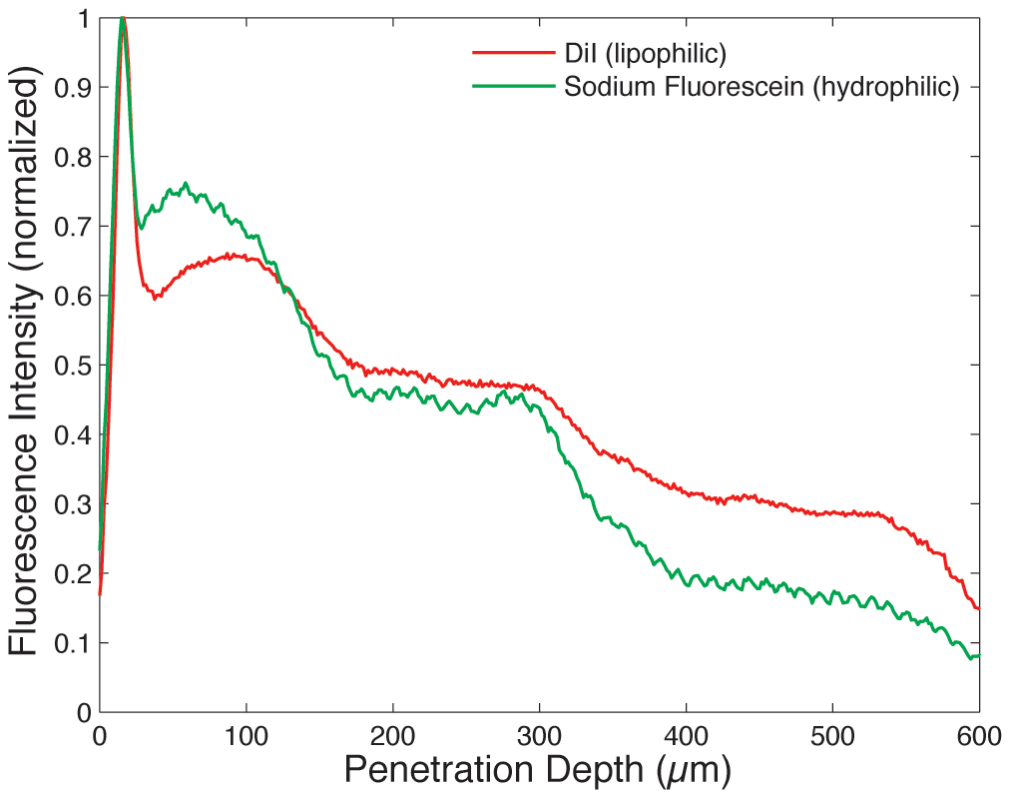
Penetration depth profiles of lipophilic and hydrophilic liposomal dyes within a tissue section following 15 min application of 31 nm PEGyated liposomes simultaneously loaded with both dyes. Each curve is representative of an average of 5 ROIs per image. A Pearson's correlation coefficient of ρ = 0.92 reveals a high degree of colocalization between the dyes.

Regardless of their transport efficiency, it has been unclear if liposomes can traverse the SC intact. Penetration of fluorescent reporter molecules may occur as a result of liposome rupture or leakage during passage through the SC, with enhanced permeation of free dye possibly resulting from interactions between liposomes and dermal lipid structures. To explore this issue for the case of the microfluidic-enabled liposomes, a combination of hydrophilic dye (SF) and lipophilic dye (DiI) were simultaneously incorporated during liposome formation into the vesicle cores and bilayers, respectively. Due to the lipid structure of the SC, diffusive transport of free hydrophilic and hydrophobic solutes is expected to vary significantly [33], [34], such that a lack of spatial correlation between the two dyes would imply that the liposomes had ruptured or leaked, allowing the hydrophilic dye (SF) to permeate through the tissue at a different rate than the lipophilic dye (DiI). Conversely, a high degree of spatial correlation would suggest the presence of intact vesicles. For the case of 31 nm liposomes, two color imaging of the exposed tissue sections reveals strong agreement between the distributions of hydrophilic (green) and lipophilic (red) signals through the SC and into the epidermis for all samples, as revealed through both the images (Fig. 5) and the dye penetration depth profiles taken through the depth of the tissue (Fig. 6). Using Pearson's correlation coefficient (ρ) as a measure of the degree of linear dependence between the spatial distributions of each dye, an average value of ρ = 0.92 was determined for the 31 nm PEGylated liposomes, indicating a high degree of correlation between the dye locations. While not conclusive, this evidence strongly suggests that the small liposomes successfully penetrate through the SC intact with minimal leakage of their cargo. Experiments performed using larger liposomes resulted in measured values of ρ = 0.81 and ρ = 0.75 for 308 nm and 105 nm liposomes, respectively. This relatively poor correlation, together with the overall lack of significant dye penetration (Fig. 3), indicates that some degree of vesicle degradation and free dye diffusion occurs for these larger liposomes.

In conclusion, we have leveraged a microfluidic liposome synthesis technique to evaluate size-dependent transdermal delivery of liposomes through *ex vivo* porcine tissues. Compared to larger vesicles, where dye penetration across the SC is presumed to occur primarily through a combination of vesicle rupture and transport along follicular pathways, the smaller 31 nm diameter and 41 nm diameter liposomes traverse and transport their intra-liposomal contents across the full surface of the SC and into deep dermal tissues, with penetration depths of at least several hundred micrometers observed with a short 15 min incubation. Multicolor fluorescence imaging of hydrophilic and hydrophobic dyes incorporated into the liposomes during synthesis further reveals that the smallest 31 nm liposomes are able to traverse dermal layers intact, with implications for clinical applications requiring co-delivery of therapeutic reagents with dissimilar chemical properties, nanoparticle-mediated drug release, or transport of intact nanocarriers to the bloodstream for systemic delivery. The results presented here also represent the first demonstration of passive transdermal diffusion of nanoscale, microfluidic-generated liposomes, opening the door to the use of these nanoparticles for effective delivery of lipophilic, hydrophilic, and amphipathic compounds to underlying dermal layers. The transport of nanoparticles through the SC is a matter of much debate, and the findings of the present study will require additional validation using complementary methods to confirm the size-dependent behavior described here and assess the fate of both liposomes and cargo.

## Materials and Methods

Certain commercial equipment, instruments, or materials are identified in this paper to foster understanding. Such identification does not imply recommendation or endorsement by the National Institute of Standards and Technology, nor does it imply that the materials or equipment identified are necessarily the best available for the purpose.

### Ethics Statement

This research involved Yorkshire piglets sacrificed as part of a separate parallel study approved by the Institutional Animal Care and Use Committee (IACUC) at the Children's National Medical Center. All procedures were performed in accordance with the National Institutes of Health Guide for the Care and Use of Animals in Research.

### Lipid Mixture and Hydration Buffer Preparation

Two variations of lipid mixtures were prepared to analyze the resulting penetration depth of both PEG-conjugated (PEGylated) and negatively-charged (anionic) into dermal tissue. In addition to enhancing liposome stability, PEG is commonly attached to the exterior of liposomes as a protective shield from the immune system during blood circulation [35], potentially increasing the bioavailability of PEGylated liposomes that are able to reach and enter subcutaneous capillaries after transdermal transport. For PEGylated liposomes, dimyristoylphosphatidylcholine (DMPC), cholesterol, and dipalmitoylphosphatidylethanolamine-PEG 2000 (PEG2000-PE) (Avanti Polar Lipids Inc., Alabaster, AL) were combined in chloroform (Mallinckrodt Baker Inc., Phillipsburg, NJ) at a molar ratio of 70∶25∶5. For anionic liposomes, DMPC, cholesterol (both from Avanti), and anionic surfactant dihexadecyl phosphate (DCP) (Sigma Aldrich, St. Louis, MO) were mixed in chloroform (Mallinckrodt Baker Inc.) at a molar ratio of 50∶40∶10. The lipid mixtures were prepared in glass scintillation vials then stored in a vacuum desiccator for at least 24 h for complete solvent removal. The desiccated lipid mixtures were re-dissolved in anhydrous ethanol (Sigma Aldrich) for a total lipid concentration of 40 mM. To assist in fluorescent imaging, a lipophilic membrane dye, 1,1′-dioctadecyl-3,3,3′,3′-tetramethylindocarbocyanine perchlorate (DiI-C_18_; DiI) (Life Technologies, Carlsbad, CA) was included into the lipid mixtures (1 wt%). A 10 mM phosphate buffered saline (PBS) (Sigma Aldrich) solution at pH 7.4 was used as a hydration buffer, with selected samples containing 1 mM hydrophilic sodium fluorescein salt (SF) (Sigma Aldrich) as a hydrophilic dye. All fluids (solvent and buffer) were passed through 0.22 μm filters (Millipore Corp., New Bedford, MA) before being introduced to the microfluidic device.

### Liposome synthesis and characterization

PEGylated and anionic liposomes were prepared using methods described previously [17]–[20]. Briefly, a flow-focusing microchannel network for liposome synthesis was fabricated following our previous work [36]. All microchannels in the final device were nominally 50 μm wide and 300 μm tall. The prepared lipid-ethanol solution was injected into the microfluidic device between two sheath flows of the aqueous buffer. The flow rate ratio (FRR), defined as the ratio of the volumetric flow rate of the aqueous buffer to the volumetric flow rate of the ethanol, was varied between 5–50 to produce liposomes with modal diameters ranging from 31 nm to 308 nm. Total average linear flow velocity for all FRRs was kept constant (0.125 m/s) for a total volumetric flow rate of 112 μL/min. To enable the formation of smaller vesicles, the temperature of the microfluidic device was controlled by contacting the glass slide of the device with a hot plate at 50°C throughout the entire synthesis process [37]. The resulting liposome populations were characterized for size via dynamic light scattering (Nano ZSP, Malvern Instruments Ltd., UK). Size distribution plots were generated by fitting spline curves to the binned distribution data imported from the dynamic light scattering instrument.

The microfluidic-generated liposomes contained lipophilic DiI in their bilayers and hydrophilic SF in their cores to enable fluorescence imaging of tissue penetration depth. To remove any remaining dye not incorporated into the liposomes during the synthesis process, all liposome samples were purified via size exclusion chromatography on Sephadex G-25 PD-10 columns (GE Healthcare, Piscataway, NJ) equilibrated with PBS immediately before application to the tissue. Gel filtration using the PD-10 columns provides efficient buffer exchange for removal of ethanol used in the liposome formation process, thereby preventing variations in ethanol concentration (2–16%) used for different liposome populations from affecting skin permeation experiments. Final lipid concentrations following gel filtration ranged from 0.56 mM to 4.76 mM, depending on the FRR used for liposome synthesis.

### Tissue exposure and cryosectioning

Porcine ear tissue from Yorkshire piglets (4 weeks, 5 kg) was selected due to its morphological and functional resemblance to human skin. Porcine ear skin *in vitro* has shown remarkably similar biophysical properties to human skin *in vivo*, particularly in terms of the diffusivity and permeability coefficient of water across the SC [38]. Studies have also indicated that porcine skin is extremely similar both structurally and chemically to its human counterpart, exhibits chemical properties which are rather consistent across different samples and stable over time at room temperature, therefore porcine skin is a valuable tool for investigating diffusion dynamics of materials with human skin [39]. One ear from each animal was removed following general anesthesia. Liposome solutions were immediately applied in 50 μL aliquots for each size in different locations on the outside of the ear, resulting in spot areas ranging from 0.25–0.5 cm^2^, and incubated for 15 min at room temperature. This exposure method was chosen over the use of a perfusion cell since the focus of this study is on short-term SC transport rather than long-term behavior of the nanoparticles within the dermis. For the characterization of size-dependent transport, all liposome solutions covering the full range of size distributions were deposited on ears from a single animal to minimize the influence of tissue morphology variations between animals. Different animals were used for each set of experiments characterizing PEGylated liposome transport, anionic liposome transport, and co-distribution of lipophilic and hydrophilic dyes. Following incubation, the ear tissue was placed in a plastic petri dish and frozen. The frozen tissue was bulk sectioned, embedded using Tissue-Tek Cryo-OCT compound (Fisher Scientific, Pittsburgh, PA), and frozen at −80°C. The frozen tissue blocks were then sectioned into smaller slices, nominally 30 μm thick and revealing dermal tissues at least 300 μm from the surface, using a HM550 cryostat microtome (Richard Allan Scientific, Kalamazoo, MI) and placed onto gelatin-treated glass slides for imaging. Sections were procured from the tissue directly beneath each of the applied liposome volumes, with the plane of each section aligned through the center of its corresponding droplet. Sectioning was performed with the blade oriented perpendicular to the skin surface and the blade path in the direction of the SC to prevent artifacts that could result from mechanical displacement of liposomes, dye, or tissue normal to the SC layer.

### Fluorescence microscopy and image processing

The 30 μm thick tissue sections were imaged using a TE-2000 S inverted epifluorescence microscope (Nikon, Melville, NY). Brightfield images and fluorescence images at 528 nm–553 nm (green filter; DiI) and 465 nm–495 nm (blue filter; SF) excitation wavelengths were acquired and overlaid to confirm and evaluate the extent of liposome penetration into the dermal tissue and to assess colocalization of the lipophilic and hydrophilic dyes.

ImageJ software (National Institutes of Health, Bethesda, MD) was used to analyze the images. Fluorescence intensity profiles were extracted using 10 μm wide regions of interest (ROIs), with data from multiple ROIs combined to generate quantitative profiles of liposome penetration depth. The intensity data was averaged across 5 ROIs per sample, then normalized to peak intensity and aligned to reveal the average fluorescence signal seen within each tissue sample below the SC. Dye colocalization was analyzed using the JACoP plugin with ImageJ [40]. In all experiments, image analysis was performed independently for each dye.
